# Impact of low-dose computed tomography for lung cancer screening on lung cancer surgical volume: The urgent need in health workforce education and training

**DOI:** 10.1097/MD.0000000000026901

**Published:** 2021-08-13

**Authors:** Yi-Chi Hung, En-Kuei Tang, Yun-Ju Wu, Chen-Jung Chang, Fu-Zong Wu

**Affiliations:** a Laboratory of Tissue-Engineering, Department of Medical Imaging and Radiological Sciences, Central Taiwan University of Science and Technology, Taichung, Taiwan; b Department of Medical Imaging and Radiology, Shu-Zen Junior College of Medicine and Management, Kaohsiung, Taiwan; c Department of Surgery, Kaohsiung Veterans General Hospital, Kaohsiung, Taiwan; d Department of Medical Education and Research, Kaohsiung Veterans General Hospital, Kaohsiung, Taiwan; e Department of Radiology, Kaohsiung Veterans General Hospital, Kaohsiung, Taiwan; f Faculty of Medicine, School of Medicine, Institute of Clinical Medicine, National Yang Ming Chiao Tung University, Taipei, Taiwan.

**Keywords:** low-dose computed tomography, lung cancer screening, surgical volume

## Abstract

This study aimed to investigate the time trend variation in the surgical volume and prognostic outcome of patients with lung cancer after the gradual prolonged implementation of a low-dose computed tomography (LDCT) lung cancer screening program.

Using the hospital-based cancer registry data on number of patients with lung cancer and deaths from 2008 to 2017, we conducted a retrospective study using a hospital-based cohort to investigate the relationship between changes in lung cancer surgical volume, the proportion of lung-sparing surgery, and prolonged prognostic outcomes after the gradual implementation of the LDCT lung cancer screening program in recent years.

From 2008 to 2017, 3251 patients were diagnosed with lung cancer according to the hospital-based cancer registry. The 5-year mortality rate decreased gradually from 83.54% to 69.44% between 2008 and 2017. The volume of total lung cancer surgical procedures and proportion of lung-sparing surgery performed gradually increased significantly from 2008 to 2017, especially from 2014 to 2017 after implementation of a large volume of LDCT lung cancer screening examinations. In conclusion, our real-world data suggest that there will be an increase in cases of operable early-stage lung cancers, which in turn will increase the surgical volume and proportion of lung-sparing surgery, after the gradual implementation of the LDCT lung cancer screening program in recent years. These findings suggest the importance of a successful national policy regarding LDCT screening programs, regulation of shortage of thoracic surgeons, thoracic radiologist workforce training positions, and education programs.

## 1 Introduction

Lung cancer is the leading cause of cancer-related deaths in Taiwan in both men and women according to the Taiwan Cancer Registry report in 2019.^[1]^ In recent years, systematic reviews and meta-analyzes have demonstrated the prolonged prognostic effect of low-dose computed tomography (LDCT) for lung cancer screening in high-risk heavy smokers, with 20% reduction in lung cancer-specific mortality compared to that in randomized concurrent controls.^[2,20]^ The prevalence of non-smoking-related lung cancer has gradually increased in Asian countries (such as China, Taiwan, Korea, and Japan) in recent years because of complex environmental and genetic factors.^[10,15,24,29]^ In Taiwan, 70.5% patients with lung adenocarcinoma were non-smokers, and most patients were diagnosed at an advanced stage with poor overall survival.^[26]^ Recent studies have demonstrated the potential impact of LDCT screening in lowering lung cancer mortality in a population with a high prevalence of non-smoking-related lung cancer.^[16,17,30]^

To date, no studies have investigated the potential impact of LDCT for lung cancer screening on the future thoracic surgical volume and proportion of lung-sparing surgery after implementation of a large volume of LDCT examinations. This retrospective study was conducted using a hospital-based cohort to investigate the relationship between changes in surgical volume and prolonged prognostic outcomes after the gradual implementation of the LDCT lung cancer screening program in recent years.

## 2 Patients and methods

### 2.1 Data collection

In Kaohsiung Veterans General Hospital, self-paid LDCT for lung cancer screening has been conducted for population-based mass screening in patients aged 40 to 80 years since 2008 because the prevalence of non-smoking related lung cancer has gradually increased. Data on the total number of LDCT screenings performed in our hospital from 2008 to 2017 were collected from the medical image database system. Data on estimates of the time trend for lung cancer incidence statistics, mortality, stage distribution, and surgical volumes were collected from the hospital-based lung cancer registry database from 2008 to 2017.

According to previous studies, gradual implementation of the LDCT lung screening program could lead to a remarkable decrease in lung cancer mortality and a remarkable stage shift in the trend over time in this hospital-based cohort in the middle of the 7-year period.^[30]^ In this study, we aimed to investigate the long-term prolonged effect of gradual implementation of the LDCT lung screening program on lung cancer mortality rate, stage distribution, overdiagnosis monitoring, and trend in surgical volume over a 10-year period. The tumors were staged according to the American Joint Committee on Cancer sixth edition for patients diagnosed between 2008 and 2009 and the American Joint Committee on Cancer seventh edition for patients diagnosed between 2010 and 2017.^[25]^ The study protocol was approved by the Institutional Review Board of Kaohsiung Veterans General Hospital (VGHKS19-CT2–09). The requirement for informed consent was waived by the institutional review board owing to the retrospective study design.

### 2.2 Statistical analysis

Descriptive statistics was calculated. Continuous data are presented as mean ± standard deviation, and categorical data are presented as frequencies with percentages. Statistical estimates were used to graphically display trends over time. Descriptive statistics on lung cancer in the database were calculated for each year from 2008 to 2017. Correlations between the 2 quantitative variables and trends in LDCT volume and descriptive statistics in the lung cancer registry database over time were analyzed using Pearson's correlations. Statistical significance was set at *P* < .05, and SPSS software (version 18; IBM, Armonk, NY, USA) was used for all statistical analyzes.

## 3 Results

Table 1 shows the annual number of LDCT screenings performed and the annual lung cancer statistics report from 2008 to 2017 according to the hospital-based cancer registry. From 2008 to 2017, 3251 patients were diagnosed with lung cancer in the target population from a hospital-based cohort. The annual number of LDCT screenings performed in the hospital-based cohort gradually increased from 245 in 2008 to 4912 in 2017, showing a 20-fold increase compared to baseline after implementation of large-volume LDCT examinations, especially from 2011 to 2017. From 2008 to 2017, the percentage of male patients diagnosed with lung cancer gradually declined (70.7% in 2008 to 54.4% in 2017), whereas that of female patients diagnosed with lung cancer diagnoses gradually increased (29.3% in 2008 to 45.6% in 2017). The mean age of patients diagnosed with lung cancer decreased from 69.05 ± 12.73 years in 2008 to 64.65 ± 11.87 years in 2017.

**Table 1:** Characteristics of all patients diagnosed with lung cancer between 2008 and 2017 by sex, age, and period during gradual implementation of low-dose computed tomography screening in the hospital-based cohort.

	2008	2009	2010	2011	2012	2013	2014	2015	2016	2017
Age (mean ± SD, yr)	69.05 ± 12.73	68.74 ± 12.56	67.71 ± 12.86	65.83 ± 13.75	66.12 ± 13.22	66.68 ± 12.09	66.91 ± 13.26	65.77 ± 12.38	63.76 ± 12.92	64.65 ± 11.87
Sex										
Male	222 (70.7%)	254 (69.6%)	201 (63.2%)	171 (60.6%)	200 (59.9%)	153 (54.1%)	199 (60.3%)	197 (55.5%)	186 (56%)	184 (54.4%)
Female	92 (29.3%)	111 (30.4%)	117 (36.8%)	111 (39.4%)	134 (40.1%)	130 (45.9%)	131 (39.7%)	158 (44.5%)	146 (44%)	154 (45.6%)
Never smoker	N/A	N/A	N/A	56.74%	55.39%	54.06%	49.70%	58.31%	59.64%	61.24%
Lung cancer number	314	365	318	282	334	283	330	355	332	338
AAH or AIS	0	1	0	0	1	1	6	4	12	16
Stage distribution										
Stage I	49	69	46	40	67	64	81	96	103	108
Stage II	7	11	15	18	14	14	15	23	13	13
Stage III	120	108	63	48	52	41	53	39	40	28
Stage IV	138	177	194	175	200	163	179	194	174	186
Lung cancer death	281	326	284	233	257	203	215	206	158	114
1-year mortality rate	46.11% (0.5181–0.4077)	49.32% (0.5455–0.4432)	44.89% (0.5056–0.3958)	37.59% (0.4352–0.3223)	37.13% (0.4255–0.3219)	36.41% (0.4231–0.3110)	33.07% (0.3844–0.2828)	34.72% (0.3993–0.3002)	29.84% (0.3511–0.2520)	24.20% (0.2924–0.1990)
5-year mortality rate	83.54% (0.8742–0.7920)	81.13% (0.8496–0.7696)	85.26% (0.8894–8108)	80.73% (0.8512–0.7591)	74.99% (0.7954–0.7019)	72.00% (0.7715–0.6663)	66.42% (0.7166–0.6112)			
LDCT number	245	434	497	1075	1710	1889	2254	3146	3460	4912
Total lung cancer surgical volume (number of procedures)	64	93	68	61	90	88	109	131	130	127
Lung sparing surgery volume (number of procedures)	11	18	16	14	27	31	32	49	61	69
Screened-lung cancer number	1	4	6	5	6	12	12	30	26	24

### 3.1 Temporal trend in mortality rate and stage distribution after implementation of the low-dose computed tomography lung cancer screening program

**Figure 1:**
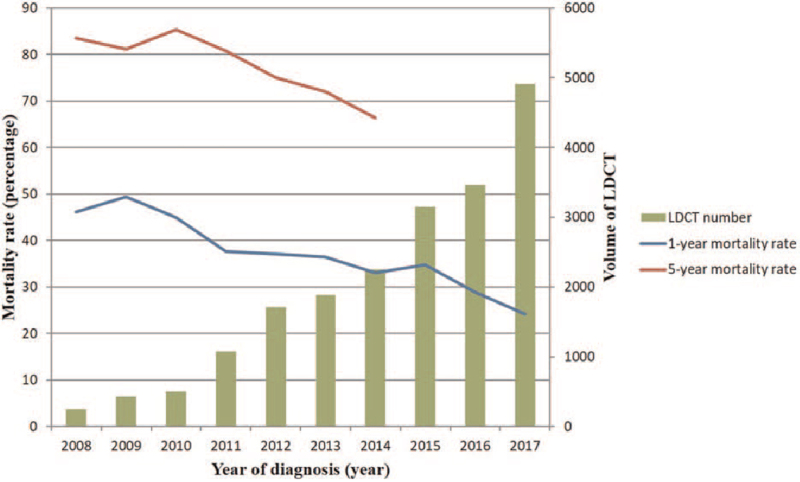
Temporal trends in prolong effect of implementation of the high-volume LDCT lung cancer screening program suggest a gradually decrease in the 1-year and 5-year lung cancer mortality between the years 2007 and 2017, especially from 2011 to 2017.

The 1-year lung cancer mortality trends revealed a gradual decrease from 46.11% in 2008 to 37.13% in 2012 and 24.20% in 2017. The 5-year lung cancer mortality trends showed a gradual decrease from 83.54% in 2008 to 74.99% in 2012 and 66.42% in 2014, as shown in Figure 1. A significant strong negative correlation was observed between the trend of mortality rate and the annual volume of LDCT screenings (1-year mortality rate, *r* = −0.941, *P* ≤ .001; 5-year mortality rate, *r* = −0.949, *P* = .001).

### 3.2 Temporal trend in stage distribution after implementation of the low-dose computed tomography lung cancer screening program

**Figure 2:**
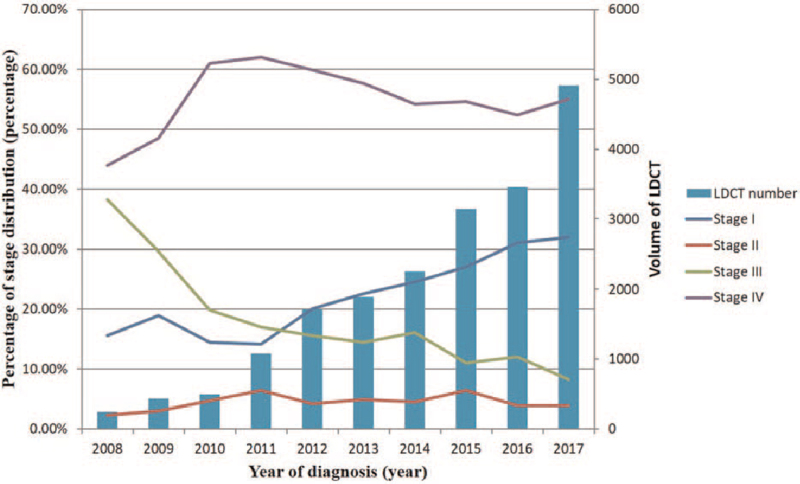
Temporal trends in prolong effect of implementation of the high-volume LDCT lung cancer screening program suggest a gradual decrease in the proportion of stage IV lung cancer with a correspondingly gradual increase in the proportion of stage I early lung cancer between the years 2007 and 2017, especially from 2011 to 2017.

Figure 2 depicts the trends in the percentage of lung cancer stage distribution in the four-stage groups from 2008 to 2017. The proportion of patients with stage IV lung cancer gradually increased from 2008 to 2011, whereas that of patients with stage III lung cancer decreased. The proportion of patients with stage IV lung cancer reached a plateau between 2010 and 2011. Between 2011 and 2017, after implementation of a large volume of LDCT examinations, a gradual decrease in the proportion of patients with stage IV lung cancer was observed, with a corresponding gradual increase in the proportion of patients with stage I lung cancer.

There was a positive correlation between the percentage of patients with stage I lung cancer and the annual volume of LDCT screenings (*r* = 0.938, *P* ≤ .001). However, no significant correlation was observed between the percentage of patients with stage IV lung cancer and the annual volume of LDCT screenings (*r* = 0.104, *P* = .776).

### 3.3 Temporal trend in overdiagnosis upon the implementation of the low-dose computed tomography lung cancer screening program

**Figure 3:**
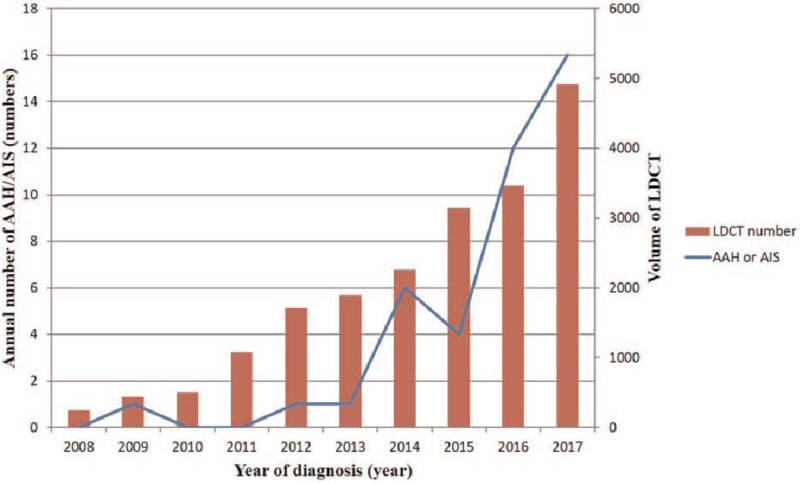
Temporal trends in prolong effect of implementation of the high-volume LDCT lung cancer screening program suggest a gradually increase in the incidence of carcinoma in situ and its precursor lesions, especially from 2014 to 2017.

For adenocarcinoma in situ and its precursor lesions, an increasing trend was observed from 2008 to 2017, especially noticeable in the last 3 or 4 years, as shown in Figure 3. The increasing trend in the last period might be due to the implementation of a large volume of LDCT examinations between 2014 and 2017. In addition, Figure 3 also shows the gradually increasing numbers of lung cancers detected during after the implementation of the large-volume LDCT examination, especially from 2013 to 2017.

### 3.4 Temporal trend in surgical volume after implementation of the low-dose computed tomography lung cancer screening program

**Figure 4:**
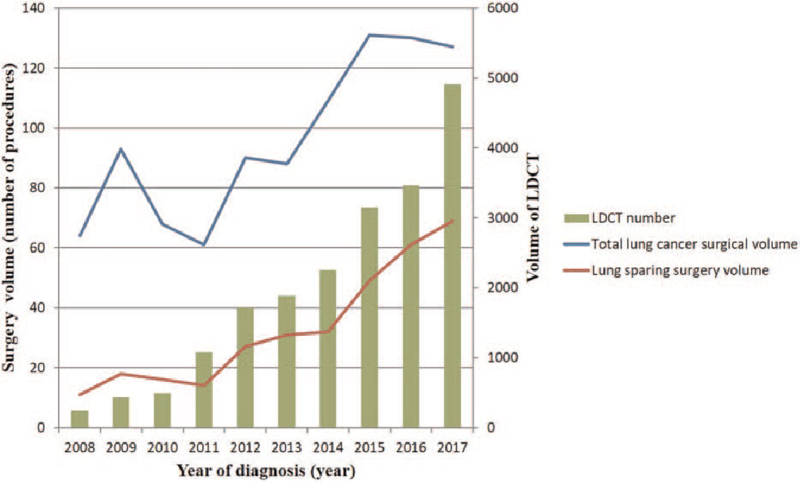
Temporal trends in prolong effect of implementation of the high-volume LDCT lung cancer screening program suggest a gradually increase in the total lung cancer surgical volume with a proportion of lung sparing surgery between the years 2007 and 2017, especially from 2015 to 2017.

To help understand past and future trends in surgical volume after implementation of the LDCT lung cancer screening program, we investigate changes in the volume of total lung cancer surgery and proportion of lung-sparing surgery from 2008 to 2017 (Fig. 4). For lung cancer surgery, there was an increasing trend over time from 2008 to 2017, especially in the last 3 or 4 years (2013/2014 to 2017).

The increasing trend in the last period may be due to an increase in the number of early-stage lung cancer cases between 2014 and 2017. Figure 4 shows the gradually increase in the number and proportion of lung-sparing surgery after the implementation of a large volume of LDCT examinations, especially from 2015 to 2017.

### 3.5 Correlation between annual temporal trends in lung cancer statistics and low-dose computed tomography volume

Pearson's correlation statistics was used to evaluate the relationship between lung cancer statistics and the annual volume of LDCT screenings (Table 2). Surgical volume, the number of patients screened for lung cancer, and the number of patients with adenocarcinoma in situ and its precursor lesions were positively and statistically correlated with the annual volume of LDCT screenings. The 1-year and 5-year mortality rates, number of lung cancer deaths, and mean age were inversely correlated with the annual volume of LDCT screenings. For lung cancer stage distribution, the proportion of patients with stage I and III lung cancer was directly correlated with the annual volume of LDCT screenings. However, the correlation between patients with stage II and stage IV lung cancer and the annual volume of LDCT screenings was not statistically significant.

**Table 2:** The correlation of a temporal trend in lung cancer characteristics with low-dose computed tomography implementation in the hospital cohort.

	LDCT number (year by year, 2008–2017)
Age (yr)	−0.840 (0.002)
Total lung cancer surgical volume (number of procedures)	0.865 (0.001)
Lung sparing surgery volume (number of procedures)	0.974 (<0.001)
Screened-lung cancer number	0.886 (0.001)
1-year mortality rate	−0.941 (<0.001)
5-year mortality rate	−0.949 (0.001)
Lung cancer death	−0.936 (<0.001)
AAH or AIS	0.905 (<0.001)
Stage I (%)	0.938 (<0.001)
Stage II (%)	0.210 (0.561)
Stage III (%)	−0.802 (0.005)
Stage IV (%)	0.104 (0.776)

## 4 Discussion

This study aimed to assess the impact of gradual implementation of the LDCT screening program on the mortality rate, surgical management, and potential overdiagnosis rate of lung cancer in a population with a high prevalence of non-smoking-related lung cancer. To the best of our knowledge, this is the first study to investigate the relationship between the trend in the annual volume of LDCT screenings and volume of lung cancer surgery based on real-world scenarios. This study reports 3 major findings. First, a decrease in lung cancer mortality rate and a shift in the stage distribution toward more early stages after prolonged implementation of the LDCT screening program were observed. Second, an increase in the total lung cancer surgical volume and the proportion of lung-sparing surgery after implementation of a large volume of LDCT examinations was observed, especially from 2015 to 2017. Third, for overdiagnosis estimation, an increasing trend in the incidence of carcinoma in situ and its precursor lesions after prolonged implementation of a large volume of LDCT examinations was observed, especially from 2014 to 2017. This study demonstrated that implementation of the LDCT screening program will lead to an increase in cases of operable early-stage lung cancers, which in turn will gradually increase the lung cancer surgical volume and the proportion of lung-sparing surgery. Our previous study has described the screened population targeting at mainly non-smoking population (age: 40–80 years, with mean age: 55.53 ± 8.02 years).^[30]^ In addition, the mean age and proportion sexes among patients with lung cancer have also changed over time, suggesting the impact of screening on the detection of lung cancer at an early stage. This study was mainly aimed at observing the yearly changes in real-world data from the lung cancer registry database. However, the causal relationship between gender difference and disease evolution is unclear. To our knowledge, this is the first real-world study to investigate the impact of these changes on lung cancer surgery volume after prolonged implementation of a large volume of LDCT examinations. However, other factors may have affected the volume of lung-sparing surgery. However, recent research has shown that nearly 80% patients with lung cancer are treated with lung-sparing surgery in the screening program. However, only 30% patients with lung cancers were treated with lung-sparing surgery in the National Lung Screening Trial in 1999.^[13]^ Our study results are in line with the findings of a previous microsimulation study showing the importance of the thoracic surgery workforce by Edwards et al.^[7]^ Our study in the real-world confirms that prolonged implementation of the LDCT screening program could add evidence of the impact of lung cancer screening on lung cancer surgical volume and the proportion of lung-sparing surgery.^[11,18]^ For early lung cancer surgical management, sublobar resections provide an anatomical functional advantage with shorter hospitalization and a faster postoperative recovery than lobectomy. Therefore, the role of sublobar resection such as anatomical segmentectomy or wedge resection is considered very important for early-stage lung cancer (<2 cm) management after prolonged implementation of the LDCT screening program.^[3,4,9,21]^

To monitor the effectiveness of the lung cancer screening program and the estimation of overdiagnosis, we analyzed the time trend in patients with adenocarcinoma in situ and its precursor lesions. Because there has been no nationwide lung cancer screening program in Taiwan, there was a very small number of cases diagnosed with carcinoma in situ; however, the annual number showed a significantly increasing trend after implementation of a large volume LDCT examinations, especially from 2014 to 2017. Overdiagnosis is an inevitable product in cancer screening, especially with prolonged implementation of a large volume of LDCT examinations.^[12]^ Therefore, active surveillance of the number of patients with carcinoma in situ for overdiagnosis estimation is mandatory for successful and effective lung cancer screening programs. Targeting high-risk populations by optimizing shared decision making for indeterminate pulmonary nodule management could provide an appropriate balance between the benefits and risks of lung cancer screening programs in a population with a high prevalence of non-smoking-related lung cancer presenting with subsolid nodules.^[6,22,23,28,32,33]^ Our previous studies and the study by Nawa et al have demonstrated that implementation of a large volume of LDCT examinations for lung cancer screening reduced the mortality rate in a population with a high prevalence of non-smoking-related lung cancer during the first 5 years (since 2011).^[16,17,30]^ Our results are in agreement with those of previous studies that showed a decrease in lung cancer mortality rate and a shift in the stage distribution toward more early stages after prolonged implementation of the LDCT screening program.^[31]^ In addition, the current research study demonstrated persistent mortality rate benefits from prolonged implementation of the LDCT screening program beyond 5 years and its impact on the 1-year and 5-year mortality after the 5-year period of implementation of a large volume of LDCT examination (2016–2017). However, other factors may have affected the temporal changes in lung cancer mortality. Among these factors, the introduction of a new target or chemotherapy therapy for lung cancer treatment has become important in recent years.

### 4.1 Strength and limitations

The main strength of this study is that it is, to our knowledge, the first major study of real-world data to investigate the impact of LDCT screening program implementation on lung cancer surgical volume. Compared with microsimulation studies, real-world evidence has the potential to provide more efficient information on efficient patient care, strategies for quality improvement, and generalizability of study results.^[5,14]^ Therefore, the research results are more realistic as a reference for the national medical policy on the shortage of thoracic surgery and the thoracic radiologist workforce. It is critically important for the success of a national policy regarding LDCT screening programs, regulation of the shortage of thoracic surgeons, thoracic radiologist workforce training positions, and education programs. Another advantage of our study design was its long study period (>10 years) to investigate the change in the trend of the mortality rate, stage distribution, and surgical volume of lung cancer after implementation of the LDCT screening program. The initial finding of this study shows that a small amount of screening does not have a significant effect on overdiagnosis in the early years. However, with the implementation of a large volume of LDCT examinations in the screening program, potential overdiagnosis and overmanagement have become increasingly important problems because improving the balance between the benefits and risks is essential for high-quality LDCT lung cancer screening programs.

The main limitation of this study is the insufficient screening coverage of the target population in this cohort. Therefore, selection bias was not minimized, leading to a substantial underestimation of overdiagnosis.^[8,19]^ Another limitation is that compared with randomized controlled trials, real-world studies could be limited by less or missing data, selection bias, and recall bias, all of which have distinct disadvantages.^[5,14]^ Therefore, further large randomized control studies with sufficient screening coverage >70% of the target population are mandatory for an efficient LDCT screening program.

In summary, the study results suggest that prolonged implementation a large-volume LDCT lung cancer screening program in this hospital-based cohort could lead to a remarkably prolonged decrease in lung cancer mortality and a remarkable stage shift in the trend over time. However, monitoring potential of overdiagnosis of precancer lesions is warranted after the implementation of a large volume of LDCT examinations. The volume of total lung cancer surgical procedures and proportion of lung-sparing surgery performed gradually increased significantly from 2008 to 2017, especially from 2014 to 2017, after implementation of a large volume of LDCT examinations. These findings suggest the importance of a successful national policy regarding LDCT screening programs, regulation of shortage of thoracic surgeons, thoracic radiologist workforce training positions, and education programs.^[27]^ There is an urgent need to promote subspecialty educational training programs for thoracic radiologists/thoracic surgeons, which can help in the lung cancer screening program.

## Acknowledgments

This study is based in part on data from the Cancer Registry Database provided by the Cancer Center of Kaohsiung Veterans General Hospital. The authors thank all doctors who responded to our screening and investigation.

## Author contributions

**Conceptualization:** Fu-Zong Wu.

**Data curation:** Yi-Chi Hung, En-Kuei Tang, Yun-Ju Wu, Chen-Jung C Chang.

**Formal analysis:** Yi-Chi Hung, En-Kuei Tang, Yun-Ju Wu, Chen-Jung C Chang.

**Investigation:** Fu-Zong Wu.

**Writing – original draft:** Fu-Zong Wu.

**Writing – review & editing:** Fu-Zong Wu.
